# Reptile Evolution and Genetics: An Overview

**DOI:** 10.3390/ani13121924

**Published:** 2023-06-08

**Authors:** Ettore Olmo

**Affiliations:** Department of Life and Environmental Sciences, Università Politecnica delle Marche via Brecce Bianche Ancona, 60121 Ancona, Italy; e.olmo@univpm.it

The study of evolution has been indissolubly linked to the study of heredity since its inception [1]. Therefore, genetic and genomic research is essential to understand the phylogenetic relationship at different taxonomic levels and to outline the main evolutionary trends.

The works in this Special Issue contribute to developing more efficient molecular methods, such as the most recent DNA sequencing techniques; isolating single chromosomes or part of them via flow sorting or microdissection; amplifying specific DNA sequences; and identifying the exact localization of these probes on mitotic chromosomes or interphase nuclei.

These methodological approaches particularly demonstrated that the genome of eukaryotes, besides functional genes, contains different types of DNA like the repetitive sequences. The most noteworthy applications included highly repetitive satellite DNAs and mobile elements (transposons and retrotransposons), whose role is not still completely understood but could play a non-negligible role in evolutionary processes [2].

Since classical karyological research evidenced an extensive variation in chromosome number and structure among species, further information gleaned from molecular biology and genome sequencing regarding chromosome structure and molecular composition confirmed that these differences are widespread and might have evolutionary consequences [3].

In 1994, Gauthier [4] proposed a cladistic definition of reptiles as a monophyletic crown group containing the so-called non-avian reptiles (turtles, lizards, snakes, and crocodiles) and birds, their common ancestors, and all their descendants. Non-avian reptiles are a very interesting group for genetic and genomic studies. They occupy a critical position in the evolution of amniotes, and their evolutionary history is well known thanks to the existing meticulous fossil records. They show a wide morphological and ecological variability with shapes adapted to broadly diverse environments. Large amounts of collected data define their biogeography, biology, and physiology and evidence certain peculiarities in their reproductive and developmental biology, such as viviparity [5] and parthenogenetic reproduction [6] in some species and the transition from strictly genetic to environmental sex determination [7]. Specific characteristics are also evident from the cytogenetic point of view, such as the different gene distribution between macro- and microchromosomes and a wide interspecific and intraspecific variability in chromosome number and morphology [8].

This Special Issue contains 12 articles advancing studies on the composition and evolution of the nuclear and mitochondrial DNAs of non-avian reptiles and of the genetic mechanisms linked to biological and evolutionary peculiarities, such as sex determination, differences in DNA methylation between various tissues, and the influence of incubation temperature on gene expression. 

One relevant paper concerns genome evolution and phylogenomic [9] and evidences the advancement of whole genome sequencing in the general framework of the karyology and composition of non-avian reptiles. This study shows that genomic resources in non-avian reptiles have now accumulated more slowly than in other amniotic groups despite the extraordinary diversity of phenotypic and genomic traits.

A survey of phylogenomic investigation shows a prevalence of whole genome sequencing, especially regarding the analysis of ultraconserved elements (UCEs). However, many other types of markers exist and are increasingly well represented, being extracted from genome assembly in silico, including some with more significant information potential than UCEs for specific investigations. 

Genome sequencing research collectively identified 139 reptilian species, providing a rich resource for in silico harvesting of information-rich markers for phylogenomics and a platform for finding the connection between genomes and phenotypic evolution. These breakthroughs could open a new era of integration of non-avian reptile comparative biology, natural history, cytogenetics, and genomics.

Karyological and genomic studies have evidenced that reptiles are a karyological heterogeneous group in which some orders and suborders exhibit characters similar to those of anamniotes, and others show similarities with homeothems. The class also presents different evolutionary trends in genome and chromosome size and composition [8]. The karyological influence on evolution can occur at different levels: chromosomal, genomic, and molecular.

In non-avian reptiles, notable differences in chromosome number and composition variability can be found between and within families. These variations seem to have had different effects on evolutionary mechanisms such as speciation [10]. Examples of these differences are evidenced in the following two research works.

A study on karyotype diversification in species of Malagasy leaf-toed geckos *Uroplatus* [11] shows that these species of gecckonid have 38 to 34 uniarmed chromosomes and tend to progressively reduce the chromosome number via the translocation of microchromosomes (especially those carrying NOR) to the telomere of macrochromosomes without changing the general morphology of the chromosomes. This evolutionary tendency towards chromosome reduction is largely shared by the gecko clade. Although the translocation to the telomeres of macrochromosomes in some species occurs, the translocation to the centromere also occurs with changes in morphology from uniarmed to biarmed. 

The content of heterochromatin in these reptiles is very limited, and in some species, putative heteromorphic sex chromosomes were found.

The situation is very different in the iguanid *Liolaemus monticola* [12]. In this species, several chromosome races differ in the number and morphology of chromosomes due to centric fission and pericentric inversions on various pairs of chromosomes. These different races are arranged in a latitudinal sequence of increasing karyotype complexity from south to north.

The existence of the different chromosome races in this lizard suggests a complex evolutionary history of chromosomal rearrangements, population isolation by barriers, and hybridization. The results of this study evidence that chromosome variation could have a relevant role in reptile evolution, especially for rich-species groups such as *Liolaemus* lizards.

Investigations at the molecular level are also of significant interest, including an extensive study on the satellite DNAs in snake heterochromatin [13], which provides information on general mechanisms of molecular evolution.

This study reports the isolation of four DNA satellite families in snake species of different families: Colubridae and Viperidae. Three of these satellites are common to species of both families, while one is typical of viperids.

Analysis using FISH and BLAST methods shows that one DNA is mainly localized at the centromere of species belonging to both families, whereas the others form clusters specific on specific chromosomes or subsets of chromosomes.

Overall, the above-mentioned results, especially those on the localization of satellite DNAs, demonstrate the conservation of these repetitive elements in snakes. These results contrast the commonly shared opinion that satellite DNAs evolve extremely quickly and are usually species or genus specific [2,14]. The situation in snakes corroborates the “library” model according to which different satellite DNA families coexist in the genome of different species, and the appearance or disappearance of some of these sequences depends on changes in copy number.

Meiosis plays an essential role in controlling variability at the chromosome and gene levels [15].

Two articles studied meiosis in squamate species. Spangenberg et al. [16] analyzed mitosis and meiosis in the common adder *Vipera berus*, which has a bimodal karyotype with 16 macrochromosomes and 22 microchromosomes, using antibodies against meiotic components such as synaptinemal complex and DNA mismatch repair proteins MLH1.

Results of this research using the high-resolution SC karyotyping technique revealed the morphology of microchromosomes and differences in the dynamics of bivalent assembly between macro- and microchromosomes during meiotic prophase for the first time. Immunostaining of MLH1 showed that crossing over sites at pachytene is 49.5, and the number of MLH1 sites per bivalent reached 11, similar to that found in several species of Agamids. These MLH1 sites are higher in microbivalents than macrobivalents. This finding can be related to the enrichment of genes found generally in snake microchromosomes [17,18].

A second paper on meiosis is a review of meiotic chromosomes in the viviparous lizard *Zootoca vivipara* [19], which possesses female heterogamety and multiple sex chromosomes with variable W sex chromosome morphology and composition.

Multiple sex chromosomes and their change may influence meiosis and female meiotic drive, and they may play a role in reproductive isolation [20,21]. In two cryptic taxa of *Z. vivipara* with different W chromosomes, meiosis in spermatogenesis and oogenesis proceeded without disturbance. No variability in the chromosome pairing at the early stages of prophase I and no significant disturbance in chromosome segregation at the anaphase-telophase have been discovered. This suggests that there should be a factor maintaining multiple sex chromosomes, their equal transmission, and the course of meiosis in these cryptic forms of *Z. vivipara.* In this regard, it is interesting that the presence of interspersed elements and transposons in this lizard species is preferentially localized at centromeric, pericentromeric, and telomeric regions that are often of key importance for the spatial orientation of chromosomes in the nucleus and segregation during meiosis [22]. Therefore, we may assume that the specific cytogenetic and genomic composition of the W chromosomes and the SINE-Zv sequences in the peritelomeric–telomeric regions might play a role in the meiotic process and the behavior of sex chromosomes of *Z. vivipara.*

Another field of interest is the molecular mechanism of sex differentiation during embryonic development in TSD species. A study of the influence of temperature on gene expression in leopard geckos shows that temperature exposition during development modifies the expression of genes related to gonadal differentiation and those involved in different developmental pathways [23].

A different situation was found on the methylation level in the turtle *Chrysemys picta*, where gonads exhibit differential DNA methylation between males and females. However, no sexual differences can be recorded in somatic tissues. The results of this research highlight that differential DNA methylation is tissue specific and plays a role in gonadal formation, sex development, and maintenance post hatching, but not in the somatic tissues [24].

Besides studies on chromosomes and the nuclear genome, two papers investigate mitochondrial genome evolution.

One of them studied the DNA barcodes of terrapins and showed that this method is an excellent way to measure the diversity of a population. An analysis of the CO1 DNA of several Malaysian terrapins (eight geoemididae, three emididae, and one pelomedusid) provides new insight into the classification of terrapins and reveals the existence of potential cryptic species [25].

Another research article examined the evolutionary potential and phylogenetic utilities of duplicated CO1 control regions in some species of varanids [26]. Sequence analysis and phylogenetic relationship revealed that divergence between orthologous copies from different individuals was lower than the paralogous copies from the same individuals, indicating an independent evolution of the CRs. These results suggest that CO1 copies seem to have acquired concerted evolution across different species.

Promising perspectives for future studies of the evolution and genetics of reptiles derive from an extensive review of antimicrobials in snake venoms and a tentative hypothesis of the karyotype of dinosaurs.

Snakes have the relevant ability to live in different environments, resist different pathogens, and eat different prey; this could be linked to an immunity similar to that of mammals [27].

One of the major problems facing public health is the growing resistance of microbes to antibiotics, so multiple scientific approaches have been employed to find new antimicrobials with high therapeutic indexes. As a result, several natural secretions, including snake venoms, have been considered sources of bioactive compounds [28,29].

The review by Oguiura et al. [30] shows that snake venoms are rich in biomolecules that can be explored as biological tools for potential anti-inflammatory, analgesic, antitumor, and antimicrobial agents. This work also describes new beta-defensin sequences of *Sistrurus miliaris*. Another significant result obtained by Oguiura and colleagues is the advantage of using multidisciplinary approaches, including sequence phylogeny, with traditional techniques for searching for new molecules with therapeutic potential.

The paper from Griffin et al. [31] is an intriguing review on the state of the art of tentative reconstruction of dinosaurs’ karyotypes research.

The divergence between the main lineage of crown reptiles, Lepidosauromorpha (tuatara, lizards, and snakes) and Archosauromorpha (turtles, crocodiles, dinosaurs, and birds), dates back to about 250 million years ago [32]. Despite the ancient divergence time, all crown reptiles’ chromosomes and genome variability are low. In particular, most species, except crocodiles, of the two lineages have a karyotype characterized by the presence of macrochromosomes and microchromosomes [10], and recent studies suggest that this pattern was probably established about 255 years ago before the first divergence of main lineages of crown reptiles.

As no intact DNA is available from fossil dinosaurs, information about extinct dinosaurs’ karyotypes can be inferred via comparative analysis of chromosomes and genomes of several species of birds and non-avian reptiles.

One approach, based on aligning chromosome-level assemblies from extant birds, determined the most likely ancestral karyotype of all birds [33]. A similar approach was used to reproduce the diapsid ancestral karyotype [34].

Another approach using chicken chromosome painting on chromosome sets of various turtle species evidenced the synteny in macrochromosomes of birds and turtles [35,36].

All of these results show that the avian chromosome pattern remained unchanged not only in most birds but also many extinct dinosaurs with a high degree of certainty [37].

The papers from this Special Issue summarize the state of genetic and genomic studies in reptiles and highlight that reptiles are a good model for studying the genetic and molecular basis of some key moments in vertebrate evolution. However, it is clear that the information collected so far is not sufficient to delineate a complete picture and that it would be important to increase the number of the whole genome sequencings and to deepen the knowledge of the molecular bases underlying some important cytological mechanisms such as meiotic pairing and segregation, the role of repetitive DNAs on the structure of chromosomes and its variations, sex determination, and the interaction between genetic and morphological level.
